# Synergistic protective effects of a statin and an angiotensin receptor blocker for initiation and progression of atherosclerosis

**DOI:** 10.1371/journal.pone.0215604

**Published:** 2019-05-03

**Authors:** Seul-Gee Lee, Seung-Jun Lee, Nguyen Viet Phuong Thuy, Jung-Sun Kim, Jung-Jae Lee, Oh-Hyun Lee, Choong-Ki Kim, Jaewon Oh, Seil Park, Ok-Hee Lee, Se Hoon Kim, Sungha Park, Sang-Hak Lee, Sung-Jin Hong, Chul-Min Ahn, Byeong-Keuk Kim, Young-Guk Ko, Donghoon Choi, Myeong-Ki Hong, Yangsoo Jang

**Affiliations:** 1 Cardiovascular Research Institute, Yonsei University College of Medicine, Seoul, Korea; 2 Severance Integrative Research Institute for Cerebral and Cardiovascular Diseases, Yonsei University Health System, Seoul, Korea; 3 Cardiology Division, Severance Cardiovascular Hospital, Yonsei University College of Medicine, Seoul, Korea; 4 Cho Ray Hospital, HCMC, Ho Chi Minh, Vietnam; 5 Cardiovascular Product Evaluation Center, Yonsei University College of Medicine, Seoul, Korea; 6 Graduate Program in Science for Aging, Yonsei University, Seoul, Korea; 7 Department of Pathology, Yonsei University College of Medicine, Seoul, Korea; Max Delbruck Centrum fur Molekulare Medizin Berlin Buch, GERMANY

## Abstract

**Aim:**

Although the atheroprotective effects of statins and angiotensin II receptor blockers (ARBs) are well-established, little is known about their additive effects, especially during the early period of atherosclerosis. The aim of this study was to investigate whether combination of a statin and an ARB exerts synergistic anti-atherosclerotic effects, and to elucidate the mechanisms of combined effects.

**Methods:**

Atherosclerotic plaques were developed in arteries of 23 rabbits using a high-cholesterol diet (HCD) and intra-arterial balloon inflation. Rabbits received one of five different treatment strategies for 4 weeks: positive control [n = 5, HCD]; negative control [n = 3, regular chow diet]; statin [n = 5, HCD and rosuvastatin 10 mg]; ARB [n = 5, HCD and olmesartan 20 mg]; and combination [n = 5, HCD and statin+ARB].

**Results:**

Histological analysis demonstrated that development of atherosclerotic plaques was inhibited more in combination group than in statin group (P = 0.001). Although macrophage infiltration identified by RAM11 staining was not significantly different between combination and individual treatment groups (31.76±4.84% [combination] vs. 38.11±6.53% [statin; P = 0.35] or 35.14±2.87% [ARB; P = 0.62]), the relative proportion of pro-inflammatory M1-macrophages was significantly lower in combination group than in ARB group (3.20±0.47% vs. 5.20±0.78%, P = 0.02). Furthermore, M2-macrophage polarization was higher in combination group than in statin group (17.70±3.04% vs. 7.86±0.68%, P = 0.001).

**Conclusion:**

Combination treatment with a statin and an ARB produced synergistic protective effects for atherosclerosis initiation and progression, which may be attributed to modulation of macrophage characteristics in the early period of atherosclerosis.

## Introduction

Cardiovascular diseases (CVDs) are the leading cause of death worldwide [1]. They are primarily caused by atherosclerosis, a composite of complex inflammatory and immunologic responses resulting in atheromatous plaques within the lining of arteries [2–4]. Following lipid accumulation, robust inflammatory responses occur within the intima, producing endothelial injury and dysfunction [3]. During these inflammatory processes, subsets of monocytes play substantial roles in the progression of atherosclerosis and evolve into various phenotypes of macrophages, promoting or inhibiting plaque development [5]. Macrophages involved in the progression of atherosclerosis have different effects on atherosclerosis, according to their polarity. While pro-inflammatory macrophages (M1-type) are usually enriched in progressing plaques, M2-macrophages, which promote tissue repair, are more enriched in regressing plaques [6,7].

Statins are the first-line pharmacological treatment for preventing atherosclerotic plaque progression. Furthermore, statins can stabilize and even induce regression of atherosclerotic plaques through their pleotropic effects, including anti-inflammatory activity as well as lipid-lowering effects [2,8]. However, beneficial effects of statins on suppressing initiation and progression of atherosclerosis before definite plaque development, termed the early period, are not well elucidated.

Angiotensin II receptor blockers (ARBs) exert their cardioprotective and atheroprotective effects by directly inhibiting the effects of angiotensin-renin-aldosterone system on arteries, as well as by reducing blood pressure [9,10]. As with statins, the protective effects of ARB in the early period of atherosclerosis are not well understood, especially with respect to vascular inflammation.

Given that combined treatment with statins and ARBs is extremely common in clinical practice, as most patients with CVD also have dyslipidemia and/or hypertension [11,12], we investigated whether statins and ARBs possess synergistic effects in preventing the initiation and progression of atherosclerosis. To determine whether these drugs have specific anti-atherosclerotic effects, rather than indirect effects related to risk factor modification, we focused on initial effects after drug administration.

## Materials and methods

### Ethics statement

All rabbits were handled according to the Association for Assessment and Accreditation of Laboratory Animal Care International system. Animal experiments conformed to the International Guide for the Care and Use of Laboratory Animals, and experimental procedures were examined and approved by the Animal Research Committee of Yonsei University College of Medicine (Seoul, Republic of Korea: Accession number YUHS-IACUC: 2015–0065). The experimental study was approved by the Animal Welfare Act, and all animals received humane care. The Principles of Laboratory Animal Care formulated by the Institute of Laboratory Animal Resources (National Research Council, NIH Publication No. 85–23, revised 1996) were followed.

### Blood pressure measurement

Non-invasive blood pressure measurements were performed using a VET HDO monitor (Memodiagnostic MD_15/90 Pro, S + B medVET), which relies on high definition oscillatory method for rapid and sensitive measurement of pulse-wave related arterial wall oscillations. [13]

### Atherosclerotic rabbit model

Thirty-three male New Zealand white rabbits (12–13 weeks, weighing 3.0–4.0 kg) were individually housed in metal cages in rooms that were maintained with a 12:12 h light:dark cycle (light cycle from 8:00 to 20:00). Ten rabbits died during the study period, and 23 rabbits were included in the final results. Experimental protocol is shown in S1 Fig.

All rabbits were fed a high-cholesterol diet (HCD; 1% cholesterol, Dooyeol Biotech, Seoul, Korea) or regular chow diet for 1 week, and then underwent abdominal aortic and iliac endothelium denudation with an intravascular balloon catheter (3.5×15 mm or 2.75×15 mm). The rabbits were then randomized to one of five groups: positive control group (n = 5, HCD); negative control group (n = 3, regular chow diet); statin group (n = 5, HCD and rosuvastatin, 10 mg/kg); ARB group (n = 5, HCD and olmesartan, 20 mg/kg); and combination group (n = 5, HCD and statin+ARB) for 4 weeks. Feeding was performed during the light period, and drugs were mixed into each animal’s food.

### Balloon injury procedure

Rabbits were anesthetized with intravascular injections of Alfaxan (0.2 mg/kg, Careside, Seongnam, Korea) and xylazine (0.5 mg/kg, Rompun, Bayer, Leverkusen, Germany). Anesthesia was maintained with 1.5% isoflurane (Forane, JW Pharm, Seoul, Korea) and oxygen. Surgical access was achieved via the left carotid artery, through which a 6 F sheath was inserted. Heparin 600 IU was injected before catheterization. Angiography was performed of the aorta and iliac arteries, followed by over-inflation of the balloon at a balloon:artery ratio of 1.2:1.0 for two 30-s intervals. Inflation was performed in each arterial segment. Catheters were subsequently removed, and the left carotid artery was ligated. Muscle and fascia were sutured with a 3.0 absorbable suture, and neck incision was closed with a skin stapler. The protocol was previously described in detail [14].

At the end of this study, angiography and OCT examination were performed prior to euthanasia and harvesting of the abdominal aorta and iliac arteries.

### Cell culture

We used the murine macrophage cell line Raw 264.7 in this study. Cells were cultured in RPMI 1640 (Biowest, MO, USA) containing 10% fetal bovine serum (Biowest), 10% non-essential amino acid (Gibco, Carlsbad, CA, USA), 1% 2-mercaptoethanol (Gibco), and 10% penicillin (Gibco). Cells were maintained at 37°C in humidified air with 5% carbon dioxide. They were washed twice with pH 7.4 phosphate-buffered saline (PBS; Gibco) before treatment. Cells were incubated with 0.1 μg lipopolysaccharide (LPS) for 4 h, followed by incubation with 2 mM ATP for 2 h before treatment with 10 μM rosuvastatin (Sigma Aldrich, St. Louis, MO, USA) and/or 10 μM olmesartan (Sigma Aldrich). The dose of each drug was based on the results of previous studies [15–18].

### Biochemical studies

Blood samples were collected for measurements of serum total cholesterol (TC), triglyceride (TG), low-density lipoprotein (LDL) cholesterol, and high-density lipoprotein (HDL) cholesterol at baseline and just before animal sacrifice. Serum samples were obtained by centrifugation (10 min, 1500 rpm, 4°C). DRI-CHEM 4000i (Fujifilm, Tokyo, Japan) and LDL cholesterol kit (Crystal Chem, Chicago, IL, USA) were used to obtain measurements.

### Quantitative coronary angiography

Quantitative coronary angiography was performed at baseline and just before animal sacrifice, using a quantitative coronary angiographic system in a core laboratory (Cardiovascular Research Center, Seoul, Korea). A guiding catheter was used for calibration, and reference vessel diameter (RD) and percent diameter stenosis (%DS) were measured.

### Optical coherence tomography imaging and matching with histology

Arteries were visualized using C7-XR imaging systems (LightLab Imaging, Inc., St. Jude Medical, Little Canada, MN, USA). OCT acquisition was obtained with wire pullback at 20 mm/s, and images were produced at 100 frames/s. Contrast was infused through the guiding catheter at a rate of 5 mL/s for 3 s. All OCT images were analyzed by experts at a core laboratory (Cardiovascular Research Center, Seoul, Korea). A 0.019-in optic imaging probe was used for calibration, and vessel area, lumen area, plaque area, and percent area stenosis were measured. Histological images were matched with the corresponding OCT pullback lesion segments based on anatomical features (e.g., luminal shape, neointimal thickness, presence of neovascularization), proximity to side branches, and distance from the injury balloon edge to the location of histology sample.

### Histological and immunohistochemical analysis

After constant perfusion, arteries were fixed in 10% normal buffered formalin for 24 h. Vessel sections 4 mm in length were placed in a single cassette and embedded in paraffin blocks. Sections were cut using a microtome to 4-micron thickness, and mounted on microscope slides (Muto Pure Chemicals, Tokyo, Japan). The slides were stained with hematoxylin and eosin (H&E), Masson’s trichrome, and Sirius red. For Oil Red O lipid staining, 1-mm length frozen specimens were cut using a microtome to a thickness of 10 microns.

In addition, tissue sections were stained immunologically with a mouse monoclonal anti-rabbit macrophage antibody RAM11 (DAKO, Santa Clara, CA, USA), as well antibodies for tumor necrosis factor (TNF)-α (ABCAM, Cambridge, MA, USA), cluster of differentiation (CD)163 (NovusBio, Littleton, CO, USA), and CD206 (Biorbyt, Cambridge, United Kingdom). Using a general immunohistochemistry (IHC) protocol, changes in smooth muscle actin (SMA) were evaluated with anti-SMA antibody (ABCAM, Cambridge, MA, USA).

Digital images of the vessels were obtained using Leica SCN400 (Wetzlar, Germany), and histomorphometry was performed using Leica Application Suite (LAS) 4.2 (Leica, Wetzlar, Germany). External elastic lamina (EEL), internal elastic lamina (IEL), and lumen were measured using Image J program (National Institutes of Health, Bethesda, MD, USA). The following parameters were calculated: media = EEL–IEL; intima area = IEL–lumen; and plaque = media + intima area.

### Immunofluorescence confocal microscopy

After the vessels were embedded in paraffin blocks, 4-micron sections were cut using a microtome and mounted on microscope slides. After washing with 1% PBS, slides were incubated overnight at 4°C with the primary antibody: anti-CD68 (Thermo Scientific, Long Beach, NY, USA), inducible nitric oxide synthase (iNOS; Santa Cruz Biotechnology, Santa Cruz, CA, USA) for M1-macrophages, or arginase-1 (Arg-1; Santa Cruz Biotechnology) for M2-macrophages. Slides were then washed in 1% PBS and incubated for 1 h in a dark room with the secondary antibody: fluorescein isothiocyanate–conjugated donkey anti-mouse IgG, phycoerythrin–conjugated goat anti-rabbit IgG, or Texas Red–conjugated goat anti-goat IgG (Santa Cruz Biotechnology). After slides were washed in 1% PBS for 10 min, nuclei were stained using 4′,6-diamidino-2-phenylindole (DAPI) (ImmunoBioScience, Mukilteo, WA, USA). Slides were then mounted with Fluoroshield, and analyzed using confocal microscopy (LSM700 system; Carl Zeiss Inc, Jena, Germany).

### Reverse transcription–polymerase chain reaction analysis

Reverse transcription into complementary DNA was performed using Quantitect Reverse Transcription (QIAGEN, Hilden, Germany) and PCR system. PCR was performed using AccuPower PCR Premix (Bioneer, Daejeon, Korea) and amfiEco Taq DNA Polymerase (GenDEPOT, Barker, TX, USA).

The following rabbit primers were used: RAGE, forward 5’-TGG ATC CTG CCT CTG AAC TC-3’ and reverse 5’-CTC CTG GTC TGC TCC TTC AC-3’; TNF-α, forward 5’-ATG GTC ACC CTC AGA TCA-3’ and reverse 5’-CTG AAG AGA ACC TGG GAG-3’; iNOS, forward 5’-CCC TGG AGG TTT CTG TTC AA-3’ and reverse 5’-GTC ATC TTG GTG CTT GCT GA-3’; Arg-1, forward 5’-AAC CCA TCT CTG GGG AAA AC-3’ and reverse 5’-GTC AAT TGG CTT GTG ATT GC-3’; HMG CoA reductase, forward 5’-GGG TTC GCA GTG ATA AAG GA-3’ and reverse 5’-CTG CCA GAA TCT GCA TTT CA-3’; AGTR1, forward 5’- TTT GGG AAC AGC TTG GCG GT-3’ and reverse 5’- GCC AGC CAG CAG CCA AAT AA-3’; GAPDH, forward 5’-AGG TCA TCC ACG ACC ACT TC-3’ and reverse 5’-GTG AGT TTC CCG TTC AGC TC-3’.

The following Raw264.7 primers were used: β-actin, forward 5’-CCT CTA TGC CAA CAC AGT-3’ and reverse 5’-AGC CAC CAA TCC ACA CAG-3’; IL-1β, forward 5’-ATG GCA ACT GTT CCT GAA CTC-3’ and reverse 5’-TTA GGA AGA CAC AGA TTC CAT GG-3’; IL-6, forward 5’-CTG CAA GAG ACT TCC ATC CAG-3’ and reverse 5’-GAG TGG TAT AGA CAG GTC TGT TGG-3’; TNF-α, forward 5’-TAC TGA ACT TCG GGG TGA TTG GTC C-3’ and reverse 5’-CAG CCT TGT CCC TTG AAG AGA ACC-3’; iNOS, forward 5’-CAC AAG GCC ACA TCG GAT TT-3’ and reverse 5’-TCA ATG GCA TGA GGC AGG AG-3’; Arg-1, forward 5’-AAC ACG GCA GTG GCT TTA ACC-3’ and reverse 5’-GGT TTT CAT CTG GCG CAT TC-3’. PCR products were analyzed on 1.5% agarose gels by comparison to GAPDH and β-actin mRNA.

### Western blot analysis

Western blot analysis was performed as previously described [14]. The primary antibodies were diluted with 5% bovine serum albumin. Horseradish peroxidase–conjugated secondary antibody was used.

### Statistical analysis

Data are expressed as mean±SEM. Statistical analyses were performed using SPSS v23.0 (SPSS Inc., Chicago, IL, USA). Continuous variables were compared using one-way analysis of variance for three or more groups. P values less than 0.05 were considered statistically significant.

## Results

### Changes in serum cholesterol levels

There were no differences in initial serum TC or LDL cholesterol levels among the five rabbit groups. After 4 weeks of an HCD, serum TC was 1372.80±304.15 mg/dL in positive control group, 995.60±151.13 mg/dL in statin group (p = 0.21 vs. positive control), and 971.80±184.47 mg/dL in combination group (p = 0.18 vs. positive control). Serum LDL cholesterol levels were also not significantly different between the groups at 4 weeks: positive control, 1459.64±245.74 mg/dL; statin, 917.88±143.53 mg/dL (p = 0.07 vs. positive control); and combination, 891.68±172.10 mg/dL (p = 0.06 vs. positive control). Although statin-treated groups showed trends toward lower TC and LDL cholesterol levels compared to positive control group, there were no significant differences between positive control group and any other treatment groups. These results are shown in S1 Table.

### Drug treatment effects identified by coronary angiography and optical coherence tomography

We sought to investigate whether administration of a statin and/or an ARB inhibited the initiation and progression of atherosclerosis in coronary arteries. Our preliminary experiment revealed Olmesartan induced about 5 mmHg reduction in SBP (S2 Table). Four weeks after HCD, %DS of the coronary arteries was measured by quantitative angiography (Table 1).

**Table 1:** Morphological parameters obtained by coronary angiography and optical coherence tomography.

	Negative Control (n = 3)	Positive Control (n = 5)	Statin (n = 5)	ARB (n = 5)	Statin+ARB (n = 5)
QCA					
RD, mm	2.69±0.19*	2.02±0.17	1.93±0.12	2.19±0.16	2.02±0.13
Diameter stenosis, %	3.90±0.30*	22.99±1.13	16.31±1.24*	13.59±1.14*	11.36±0.31*,†
OCT					
Vessel area, mm^2^	6.48±0.90	5.88±0.76	6.69±0.89	6.14±1.11	5.75±0.87
Lumen area, mm^2^	5.79±0.88	3.62±0.61	5.09±0.73	4.88±0.97	4.55±0.74
Plaque, mm^2^	0.69±0.08*	2.26±0.23	1.60±0.22*	1.26±0.16*	1.19±0.13*
Area stenosis, %	10.0±1.6*	42.7±3.5	25.4±2.4*	24.1±2.8*	22.8±1.3*

Values are mean±SEM.

* P<0.05 vs. positive control group and

† P<0.05 vs. statin group. ARB, angiotensin II receptor blocker; I/P, intima/plaque ratio; MLD, minimal luminal diameter; OCT, optical coherence tomography; QCA, quantitative coronary angiography; RD, reference diameter.

**Fig 1:**
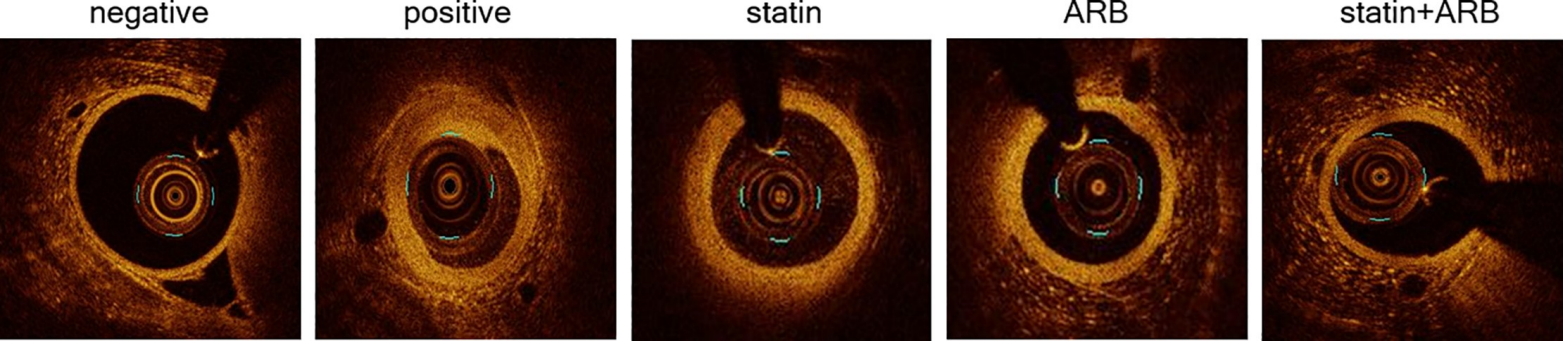
Images of optical coherence tomography (OCT). OCT images are shown for each group. Scale bars = 1 mm. ARB, angiotensin II receptor blocker.

Coronary artery stenosis was significantly mitigated in rabbits administered statin (52%), ARB (38%), or statin+ARB (62%), compared with the positive control group. Next, we compared plaque area and %AS by OCT (n = 72 images, Table 1 and Fig 1).

The results also demonstrated markedly reduced plaque in rabbits administered statin (43%), ARB (71%), or statin+ARB (78%) compared to positive control group. Taken together, the results showed that the combination of statin and ARB more potently suppressed the initiation and progression of atherosclerosis than either drug alone, although the only statistically difference was a lower %DS in statin+ARB group than in statin-only group.

### Atherosclerosis plaque reduction confirmed by histomorphological analysis

**Fig 2:**
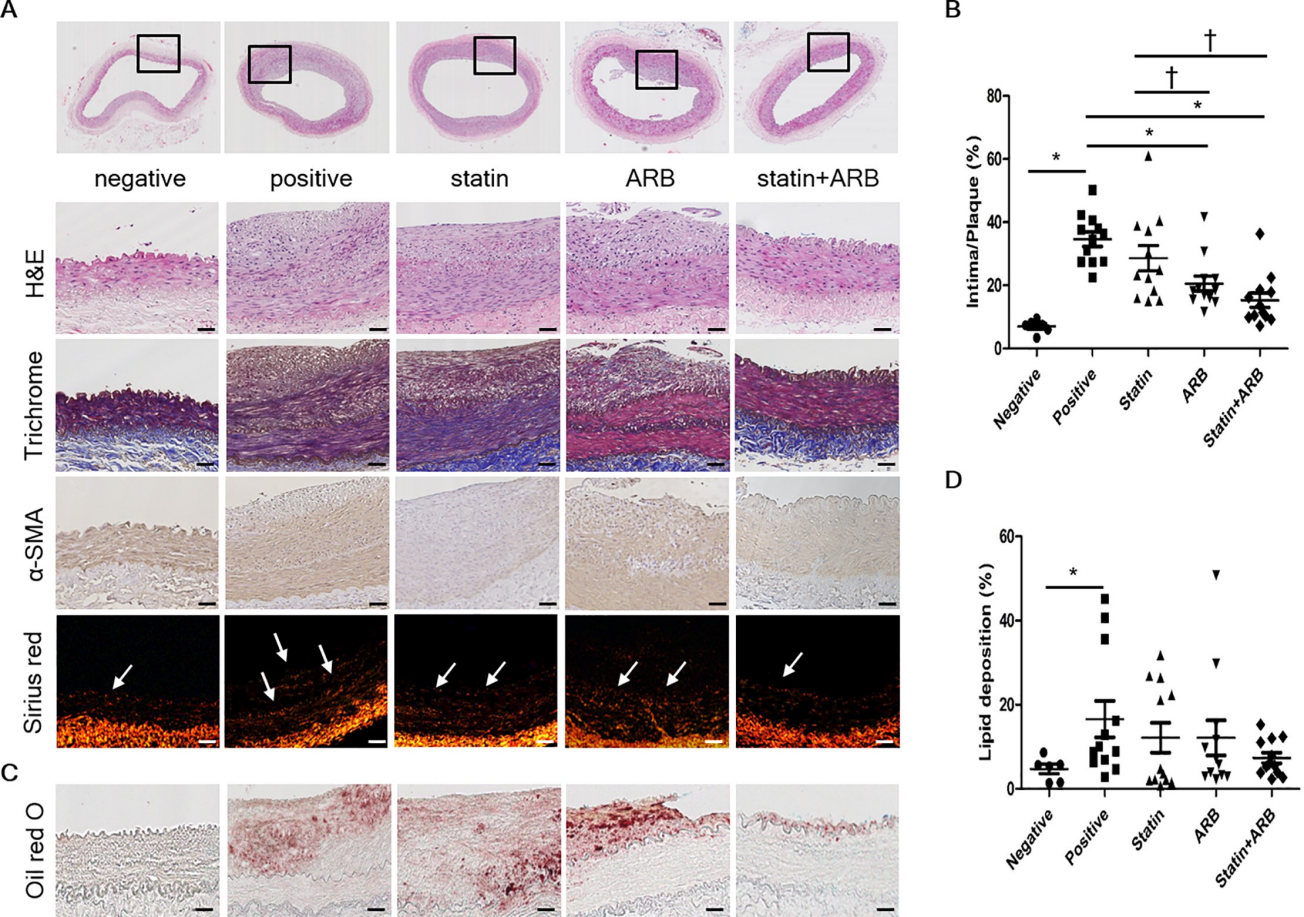
Morphologic changes by histological analysis. (A) Tissues were stained with hematoxylin & eosin (H&E) and Masson’s trichrome. Alpha-smooth muscle actin (α-SMA) content of the plaques is shown by immunohistochemical staining of α-SMA, and collagen content of the plaques is represented by Sirius red staining visualized under polarized light. (B) Intima/plaque percentage for vessels in each rabbit group. (C) Lipid deposition content of plaques analyzed by Oil Red O staining. Higher magnification of regions within the black boxes in (A). (D) Relative intensity of Oil Red O staining (lipid deposition percentage) for each group. Scale bars = 100 μm. Data are mean±SEM. * p<0.05 vs. positive control group and † p<0.05 vs. statin group. ARB, angiotensin II receptor blocker.

Representative histologic images in rabbit abdominal aorta and iliac arteries are demonstrated in Fig 2A. Morphologic analysis of H&E and Masson’s trichrome staining revealed marked reduction of intimal proliferation in combination group, compared to each treatment group. A statistically significant reduction in atheromatous plaque was observed in combination group, in comparison to positive control group (p<0.001) and ARB-only group (p = 0.001, Fig 2A and 2B). There was a trend toward less lipid accumulation (revealed by Oil Red O staining) in combination group compared to positive control group (p = 0.06, Fig 2C and 2D); however, lipid accumulation in both statin and ARB groups did not differ from that in control group (p = 0.36 for both comparisons).

### Immunohistochemical staining to assess inflammatory responses to drug treatment

**Fig 3:**
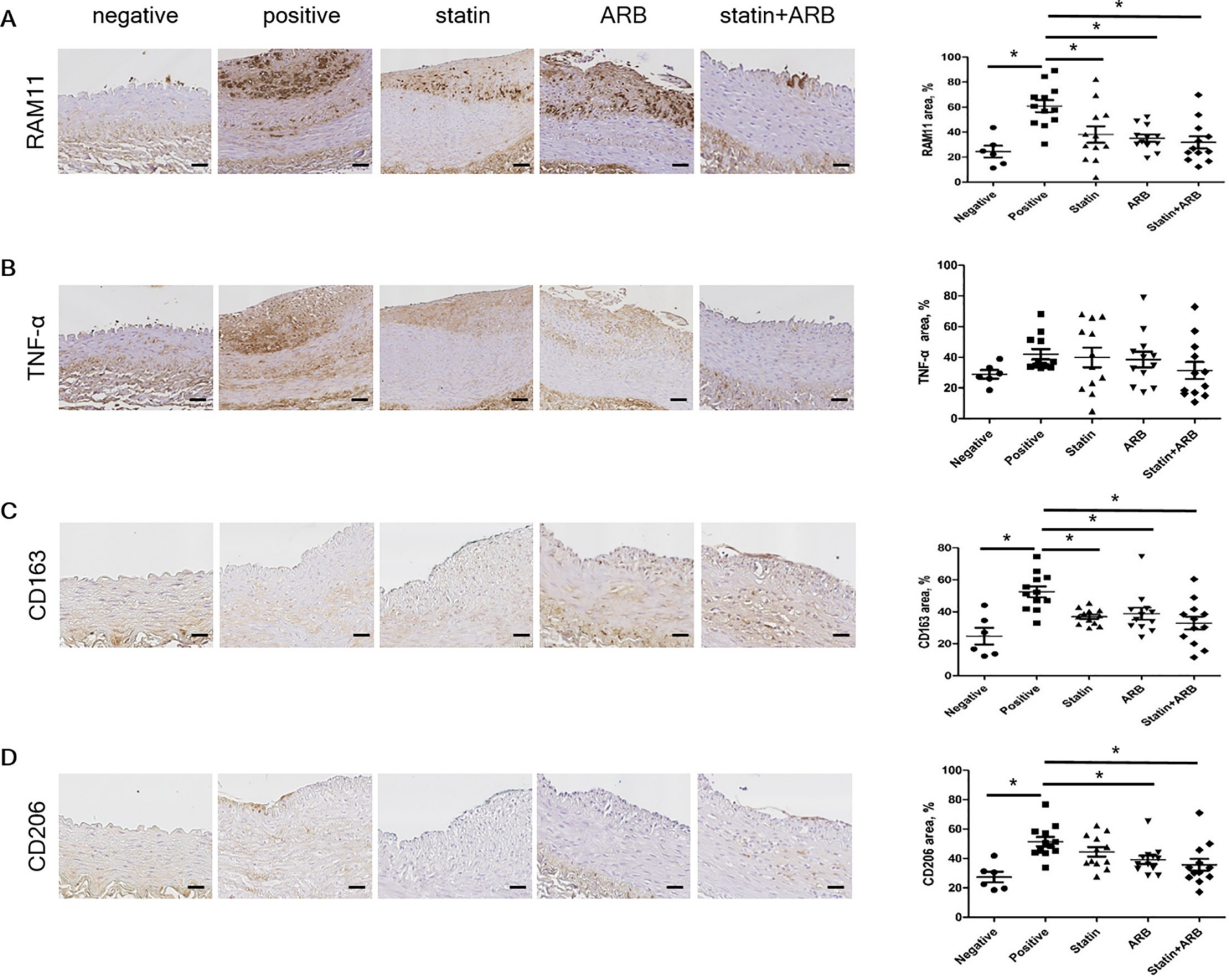
Immunohistochemical staining of macrophage markers. (A) Tissues immunologically stained with RAM11 and percentage area. (B) Tissues immunologically stained with TNF-α and percentage area. (C) Tissues immunologically stained with CD163 and percentage area. (D) Tissues immunologically stained with CD206 and percentage area. Scale bars = 100 μm. Data are mean±SEM. * p<0.05 vs. positive control group. ARB, angiotensin II receptor blocker; CD163, cluster of differentiation 163; CD206, cluster of differentiation 206; TNF-α, tumor necrosis factor-α.

To clarify the mechanisms whereby statins and ARBs synergistically suppress atherosclerosis progression, we further investigated the atherosclerotic plaques in rabbit vessels by IHC staining. Given that macrophages play a central role in the initiation and progression of atherosclerosis, we investigated whether statins and ARBs synergistically affect macrophage characteristics. Plaque macrophage content, detected by staining for anti-rabbit macrophage clone RAM11 (Fig 3A), was significantly less in combination group than in positive control group (Fig 3B). The inflammatory content of plaques, detected by staining for TNF-α (Fig 3C), tended to be less in combination group than in other groups, although the differences were not statistically significant (Fig 3D). We also used IHC to evaluate markers for macrophages observed in advanced plaques, such as CD163 (receptor of hemoglobin/haptoglobin complex) and CD206 (macrophage mannose receptor). Levels of these markers did not differ significantly between treatment groups: CD163, 32.92±4.05% in combination group vs. 36.94±1.43% in statin group (p = 0.40) and 38.86±3.77% in ARB group (p = 0.22) (Fig 3E and 3F); and CD206, 35.70±4.11% in combination group vs. 44.54±3.23% in statin group (p = 0.06) and 39.14±2.81% in ARB group (p = 0.47) (Fig 3G and 3H). Together, these results indicate that administration of a statin and/or an ARB did not affect the number of macrophages recruited to atherosclerosis plaques, even when the drugs were initiated in the early period.

### Evaluation of macrophage polarization using confocal immunofluorescence microscopy

**Fig 4:**
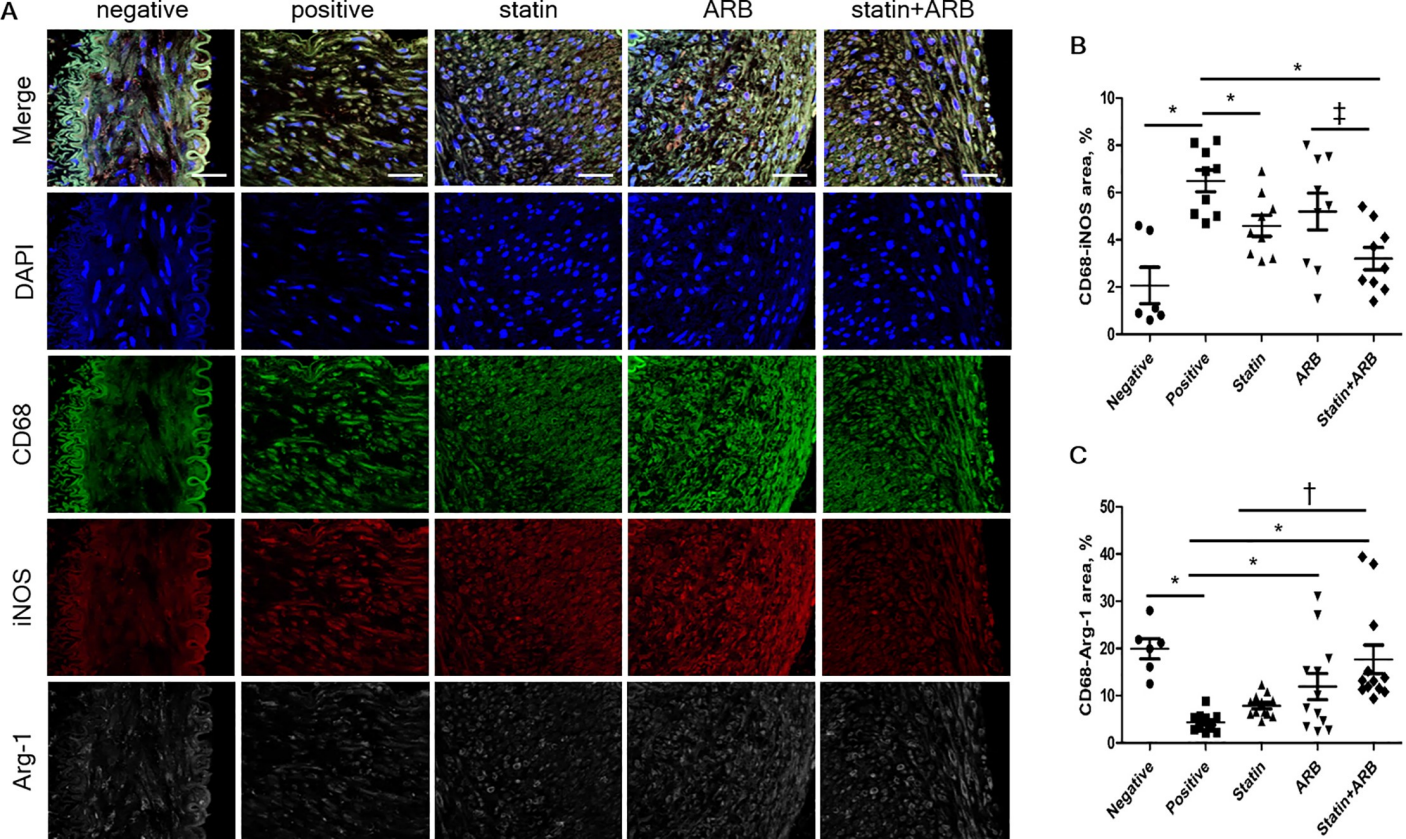
Macrophage polarization assessed by confocal immunofluorescence microscopy. (A) Confocal immunofluorescence microscopy showing CD68 (green), iNOS (red), and Arg-1 (white) localization in the same vessel. Blue represents 4′,6-diamidino-2-phenylindole staining of nuclei. (B) Relative area of CD68–iNOS staining in each group. (C) Relative area of CD68–Arg-1 staining in each group. Data are mean±SEM. * p<0.05 vs. positive control group, † p<0.05 vs. statin group, and ‡ p<0.05 vs. ARB group. ARB, angiotensin II receptor blocker; Arg-1, arginase-1; iNOS, inducible nitric oxide synthase.

We next assessed whether statins and/or ARBs affect the phenotypic characteristics of macrophages, known as macrophage polarization, in rabbit vessels. Relative area of M1-macrophage polarization, represented by iNOS staining, was significantly lower in combination group than in ARB group (3.20±0.47% vs. 5.20±0.78%, p = 0.02), and tended to be lower in combination group than in statin group (4.59±0.44%, p = 0.09). M2-macrophage polarization revealed by Arg-1 staining was significantly increased in combination group compared to either statin group (17.70±3.04% vs. 7.86±0.68%, p<0.01) or ARB group (11.92±2.76%, p = 0.05) (Fig 4A–4C).

### mRNA and protein expression of inflammatory markers in atherosclerotic plaques

**Fig 5:**
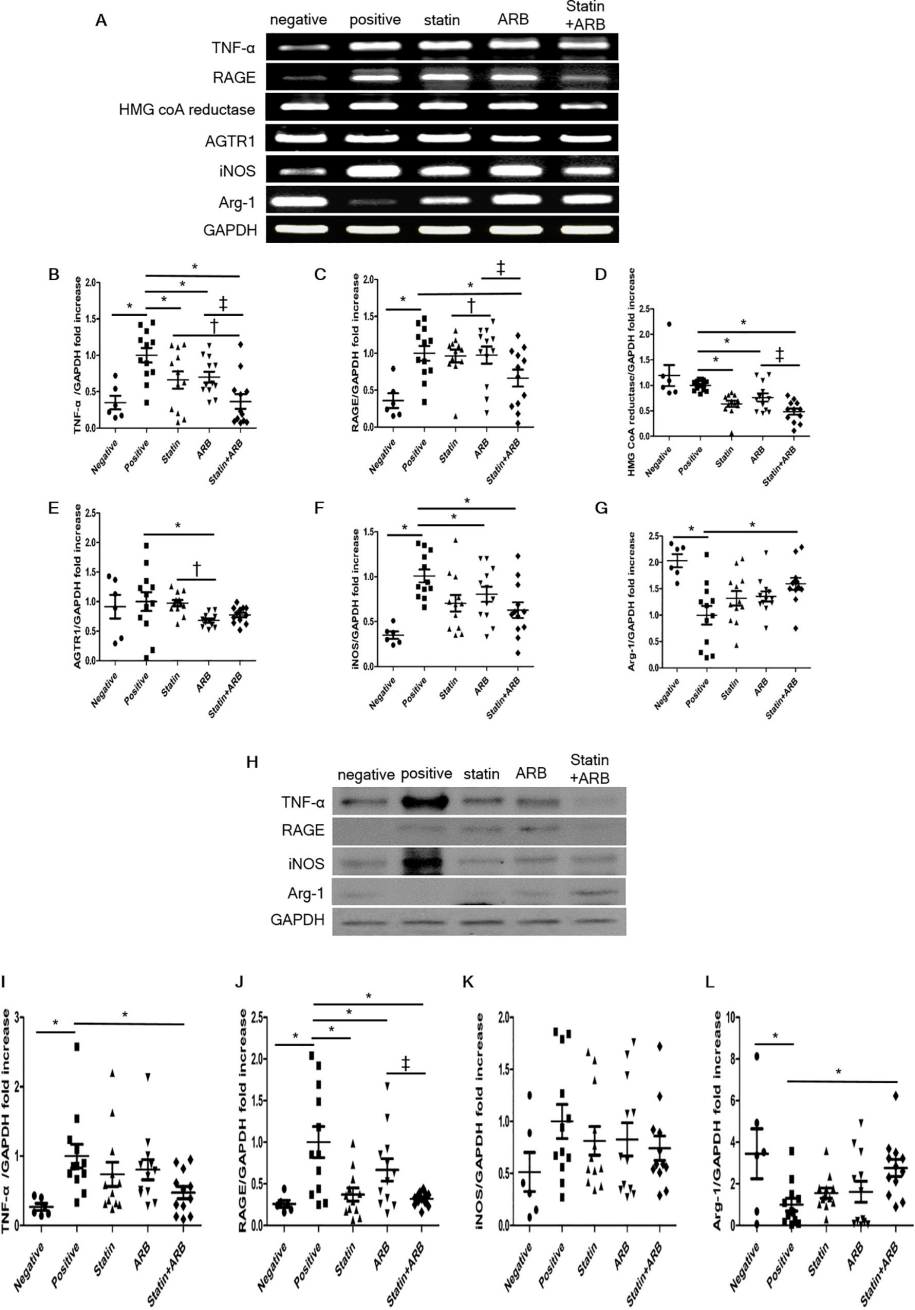
Reverse transcription–polymerase chain reaction (RT-PCR) and Western blot analysis in rabbit vessels. (A) RT-PCR expression of TNF-α, RAGE, HMG CoA reductase, iNOS, AGTR1, and Arg-1. (B-G) Comparisons of relative mRNA expression, normalized to expression of GAPDH as the housekeeping gene. (H) Western blot expression of TNF-α, RAGE, iNOS, and Arg-1. (I-L) Representative data showing protein expression, normalized to expression of GAPDH as the housekeeping gene. Data in the bar graphs are quantified ratios of the signal for TNF-α, RAGE, HMG CoA reductase, AGTR1, iNOS, and Arg-1 relative to the signal for GAPDH, presented as fold increases. Data are mean±SEM. * p<0.05 vs. positive control group, † p<0.05 vs. statin group, and ‡ p<0.05 vs. ARB group. AGTR1, angiotensin II receptor type 1; ARB, angiotensin II receptor blocker; Arg-1, arginase-1; GAPDH, glyceraldehyde 3-phosphate dehydrogenase; HMG CoA, 5-hydroxy-3-methylglutaryl–coenzyme A; iNOS, inducible nitric oxide synthase; RAGE, receptor for advanced glycation end products; TNF-α, tumor necrosis factor-α.

To identify the mechanisms of anti-atherosclerotic effects, we assessed expression of inflammatory markers in atherosclerotic plaques in rabbit vessels. Relative mRNA expression of TNF-α, receptor for advanced glycation end products (RAGE), 5-hydroxy-3-methylglutaryl–coenzyme A (HMG CoA) reductase, iNOS, angiotensin II receptor type 1 (AGTR1), and Arg-1 in each group is presented in Fig 5A.

There was a trend toward lower mRNA expression of TNF-α, RAGE, HMG CoA reductase, AGTR1, and iNOS in combination group than in other groups (Fig 5B–5F).

Conversely, mRNA expression of Arg-1, a marker of M2-macrophages, was higher in combination group than in other treatment groups (Fig 5G).

The results for protein expression of TNF-α, RAGE, iNOS, and Arg-1 were similar to the results for relative mRNA expression (Fig 5H–5L).

### Identification of drug effects *in vitro* using RAW 264.7 macrophage cell line

To determine the putative mechanisms whereby statins and ARBs synergistically polarize macrophages toward the reparative type (M2-macrophages), we analyzed changes in gene expression in the macrophage cell line Raw 264.7.

**Fig 6:**
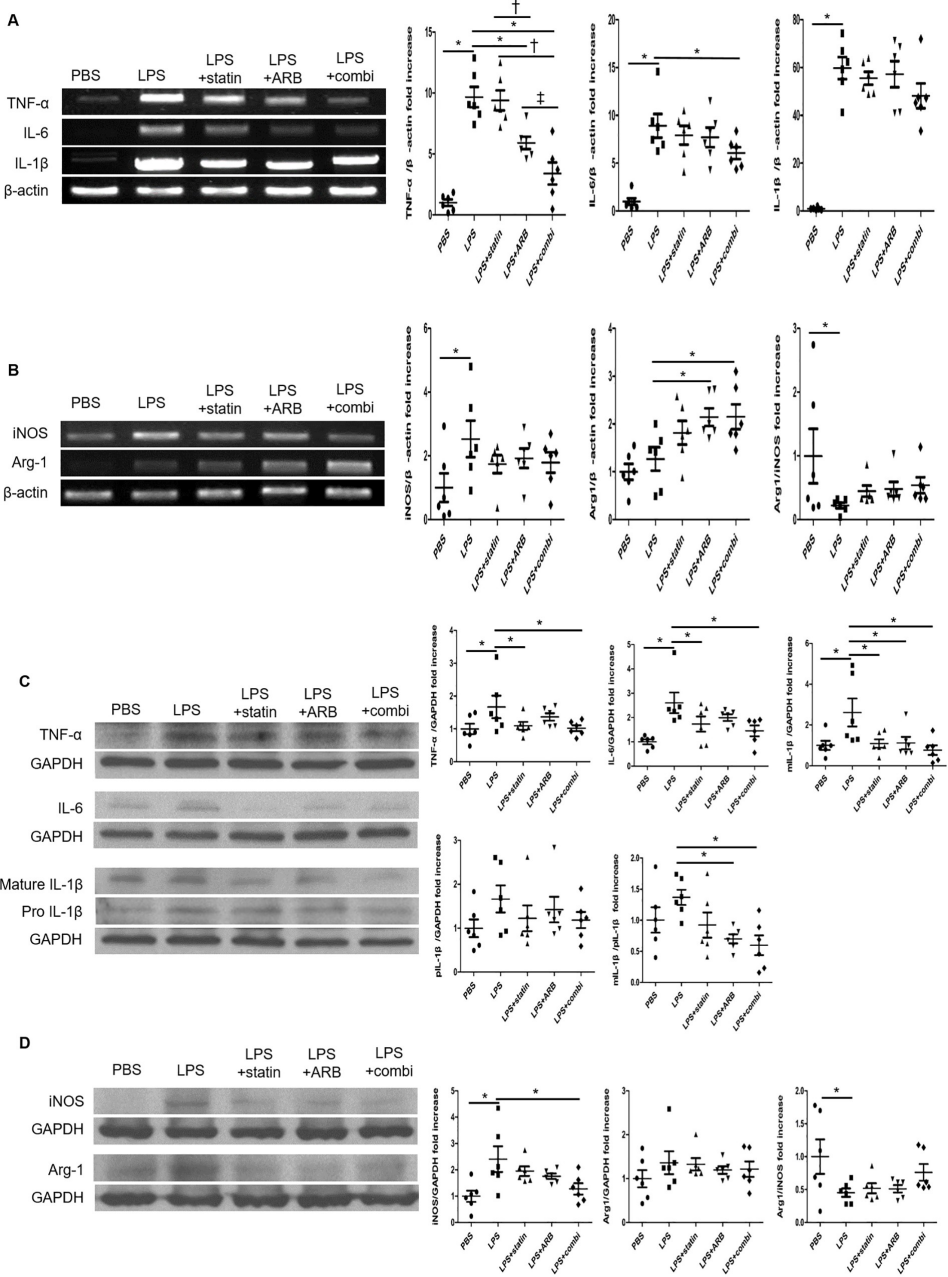
Effects of statin and ARB on the macrophage RAW 264.7 cell line, with cytokine mRNA and protein expressions. RAW 264.7 cells were stimulated with 0.1 μg LPS for 4 h, followed by 2 mM ATP for 2 h. PBS was used as the positive control. After stimulation, cells were treated for 6 h (A, C) or 24 h (B, D) with 10 μM statin, 10 μM ARB, or the combination (10 μM statin + 10 μM ARB). Data in bar graphs are quantified ratios presented as fold increases. Data represent n = 6. * p<0.05 vs. LPS group and † p<0.05 vs. LPS+statin group. ARB, angiotensin II receptor blocker; Arg-1, arginase-1; combi, combination of statin and ARB; GAPDH, glyceraldehyde 3-phosphate dehydrogenase; IL-1β, interleukin 1 beta; IL-6, interleukin 6; iNOS, inducible nitric oxide synthase; LPS, lipopolysaccharide; mIL-1β, mature IL-1β; PBS, phosphate-buffered saline; pIL-1β, pro IL-1β; TNF-α, tumor necrosis factor-α.

To mimic the inflammatory conditions of atherosclerosis, Raw 264.7 cells were first stimulated by LPS. To evaluate the anti-inflammatory effects of drug treatment, relative mRNA expression of TNF-α, interleukin (IL)-6, and IL-1β in each treatment group was determined at 6 h after drug treatment (Fig 6A). mRNA expression of inflammatory markers TNF-α and IL-6 was significantly suppressed in combination group compared to LPS-alone group (TNF-α, 9.67±0.84 fold increase in the LPS group vs. 3.40±0.91 fold increase in combination group, p<0.01; IL-6, 8.91±1.23 fold increase in LPS group vs. 6.06±0.63 fold increase in combination group, p = 0.03). IL-1β tended to be lower in combination group than in LPS group, but the differences were not statistically significant.

To assess macrophage polarization, mRNA relative expression of iNOS and Arg-1 in each treatment group at 24 h after drug treatment is presented in Fig 6B. Expression of the M1-macrophage marker iNOS tended to be lower in combination group than in LPS group. In contrast, the M2-macrophage marker Arg-1 was significantly increased in combination group compared to LPS group (2.15±0.26 fold increase in combination group vs. 1.27±0.25 fold increase in LPS group, p = 0.01).

Compared with LPS-alone group, combinational treatment of statin and ARB suppressed protein expression level of TNF-α, IL-6, and IL-1β (TNF-α, 1.67±0.34 fold increase in LPS group vs. 1.02±0.09 fold increase in combination group, p = 0.02; IL-6, 2.60±0.42 fold increase in LPS group vs. 1.45±0.22 fold increase in combination group, p<0.01; mIL-1β/pIL-1β, 1.37±0.12 fold increase in LPS group vs. 0.60±0.16 fold increase in combination group, p<0.01, Fig 6C).

An iNOS and Arg-1 protein expressions were also similar to the results for relative mRNA expression of these markers at 24 h after drug treatment (Fig 6D).

## Discussion

**Fig 7:**
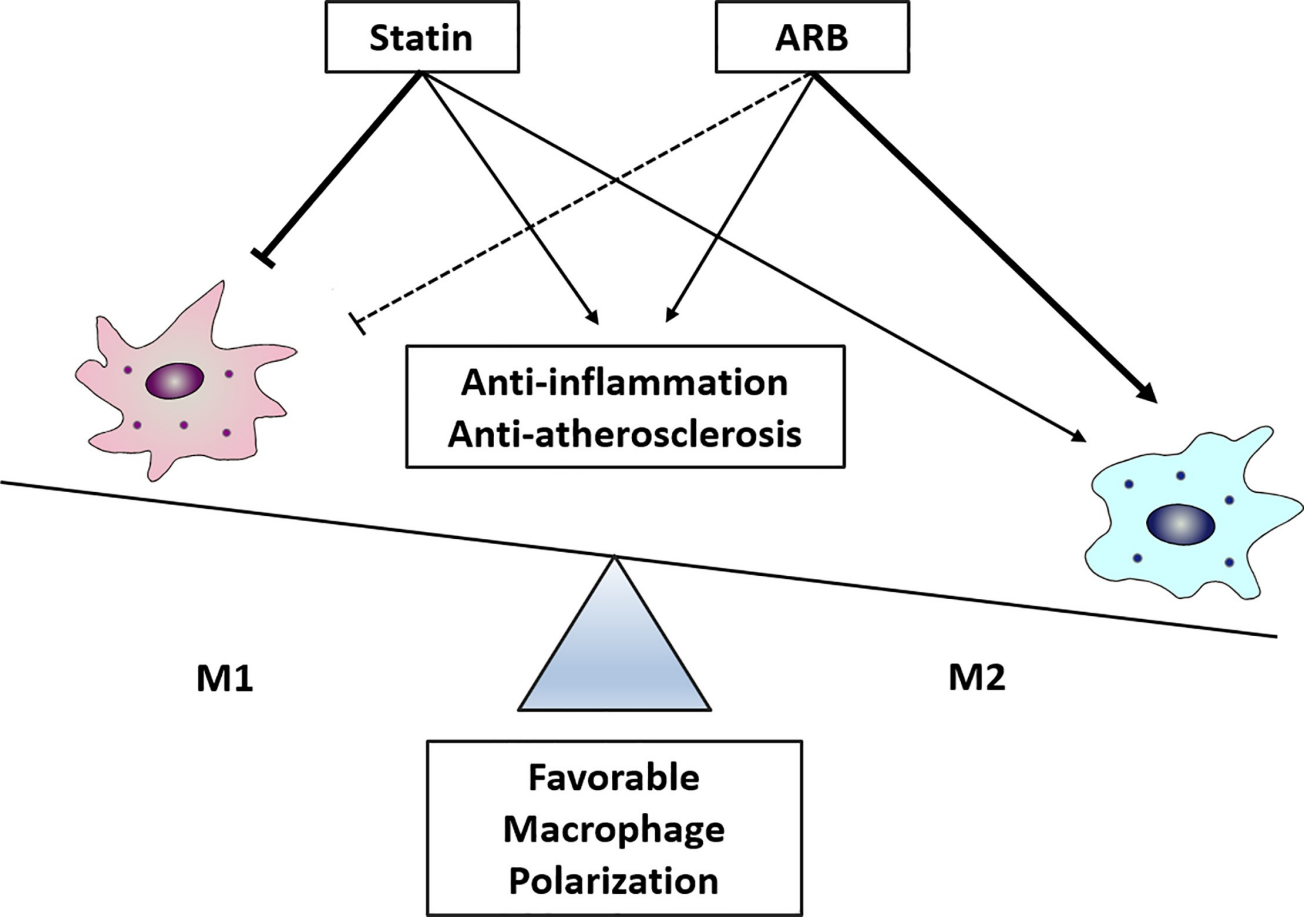
Schematic diagram showing synergistic protective effects of statin and ARB on favorable macrophage polarization.

This study demonstrated that the combination of a statin and an ARB synergistically exerts early anti-atherosclerotic effects, compared to administration of each drug separately. Further, regulation of macrophage polarization appears to play an important mechanistic role in these synergistic effects (Fig 7).

In the current study, we used an HCD-induced atherosclerosis rabbit model, which has been widely used to study the effects of drugs on atherosclerosis [19]. To evaluate acute anti-atherosclerotic effects of these drugs, rather than their well-known chronic anti-atherosclerotic effects secondary to risk factor modification, drugs were administered immediately after initiation of HCD. Under our experimental conditions, statin and/or ARB treatment did not significantly reduce serum cholesterol levels. Nevertheless, our histological analysis showed a significant decrease in atheromatous plaque ratio after statin and/or ARB treatment. These observations suggest that the combination of these drugs exhibits acute anti-arteriosclerotic effects, even before the onset of risk factor effects. In addition, imaging analyses of coronary arteries using OCT and angiography demonstrated that plaque %AS was reduced by combination treatment with a statin and an ARB. Consistent with the plaque-stabilizing effects of statins and ARBs [20,21], we confirmed the synergistic effects of these drugs on plaque characteristics by histomorphological analysis. Lipid deposition, confirmed by Oil Red O staining, was markedly decreased in combination treatment group. Furthermore, collagen accumulation and smooth muscle cell proliferation was less with combination treatment. Further, mRNA and protein expression of TNF-α, one of the most potent pro-inflammatory cytokines, which exacerbates plaque progression and induces rupture [22], was also lowest in combination treatment group. Taken together, these results suggest that combination treatment results in synergistic stabilization effects in atherosclerotic plaques.

A previous report demonstrated that a statin plus ARB markedly reduced atherosclerotic plaque lesion area by reducing RAM11 macrophage content of vascular plaques in hyperlipidemic rabbits [23]. Similarly, our IHC data showed that macrophage area, as identified by RAM11 staining, was the lowest in combined group compared to other groups. These results led us to further investigate the effects of statins and ARBs on macrophage characteristics. Macrophage balance between M1/M2 polarization is known to play an important role in the initiation and progression of atherosclerotic plaques [5,24]. It has been suggested that highly-inflammatory Ly6C^hi^ monocytes evolve into M1-macrophages in atherosclerotic lesions, which predominate in advanced vulnerable plaques and play an important role in plaque pathogenesis [7]. Conversely, M2-macrophages are associated with plaque stability because of their location within the human plaque and their intrinsic anti-inflammatory properties [24]. Further, macrophages observed near the necrotic lipid plaque core have reduced expression of Arg-1, a representative M2-macrophage marker [25]. In the current study, immunofluorescence staining for either M1-macrophage marker (iNOS) or M2-macrophage marker (Arg-1) demonstrated that combination treatment synergistically decreased iNOS area compared to ARB treatment, and increased Arg-1 area compared to statin treatment. In addition, areas of macrophages expressing CD163 or CD206, which are frequently observed in high-risk plaques [26,27], were lower in combination treatment group than in monotherapy groups, indicating that combination treatment with a statin and an ARB synergistically affects macrophage polarization, and thereby may exert beneficial effects on plaque stability. Mechanistically, both statin and ARB treatment alone showed a trend toward suppressing M1 polarization of macrophages, whereas ARB treatment alone increased M2-macrophage polarization, resulting in favorable macrophage polarization.

Along with favorable macrophage polarization, combined administration of statin and ARB reduced expression of receptors for these drugs. Previous reports have shown that statins down-regulate AGTR1 [28,29] and inhibit HMG CoA reductase [30]. Indeed, AGTR1 expression was slightly decreased by statin treatment alone, and combination drug treatment synergistically decreased AGTR1 mRNA expression. Koga and colleagues have nicely shown that AGTR1 on vascular wall cells, not bone marrow hematopoietic cells, plays a crucial role in the pathogenesis of atherosclerosis by up-regulating the expression of adhesion molecules, thus inducing monocyte recruitment [31]. This interesting result supports our claim that ARB may directly contribute to the suppression of atherosclerosis initiation and progression, as well as blood pressure reduction. Furthermore, HMC CoA reductase mRNA expression was further suppressed by combined administration of statin and ARB, compared with statin alone, which was possibly attributable to the pleiotropic effects of ARBs [32]. Using genetically modified mice, Sakai and colleagues have shown that HMG CoA reductase contributes to atherosclerosis by promoting migratory activity of monocytes/macrophage into atherosclerotic plaques through regulating the expression of small GTPase proteins, such as RhoA (RAS homolog family member A) [33]. This interesting finding that highlighted the central role of HMG CoA reductase in atherosclerosis, again supports our finding that statin and ARB synergistically suppresses atherosclerosis by suppressing HMG CoA reductase expression.

The main limitation of this study was its short experimental period, which resulted in negligible lipid-lowering effects. However, this allowed us to specifically assess the initial effects of treatment with a statin and an ARB. Also, a limited number of rabbits were available for this study, which prohibited statistical significance from being achieved in some experiments. Nevertheless, distinct trends were observed supporting the synergistic effects of statins and ARBs.

In conclusion, combined administration of a statin and an ARB exerted synergistic acute anti-atherosclerotic effects by reducing plaque burden, as confirmed by live-imaging analyses, as well as histological analyses. Mechanistically, co-administration of these agents polarized macrophages toward more reparative M2-macrophages, potentially leading to atherosclerosis suppression. These findings suggest that, in patients with coronary artery disease in a clinical setting, initiating concomitant administration of the two drugs at disease onset may lead to superior clinical outcomes.

## Supporting information

S1 Fig Schematic design of the study protocol. After 1 week of high-cholesterol diet, balloon injury was induced in the abdominal aortic and iliac arteries. Rabbits then received one of three drug treatments, depending on their group assignment, for 4 weeks. Blood was collected before the high-cholesterol diet and immediately before sacrifice at the end of week 5. Optical coherence tomography was assessed at the end of the study, after which the rabbits were euthanized for harvesting of their injured vessels.
(TIF)

S1 File Raw data for each analysis. All values for each experiment are included in this supplementary data file.
(XLSX)

S1 Table Biochemical analysis of plasma cholesterol concentration. Values are mean±SEM. * p<0.05 vs. positive control group. ARB, angiotensin II receptor blocker; HDL, high-density lipoprotein cholesterol; LDL, low-density lipoprotein cholesterol; TC, total cholesterol; TG, triglyceride.
(DOCX)

S2 Table Blood pressure lowering effect of Olmesartan. Blood pressure was measured at baseline and 1-week after treatment with 20 mg/kg/day of Olmesartan in rabbits (n = 5). Values are mean±SEM.
(DOCX)
