# Supplementary figures and images for: Synergistic protective effects of a statin and an angiotensin receptor blocker for initiation and progression of atherosclerosis
Source: PLoS One. 2019 May 3;14(5):e0215604. doi: 10.1371/journal.pone.0215604 (PMC6499436; doi:10.1371/journal.pone.0215604)

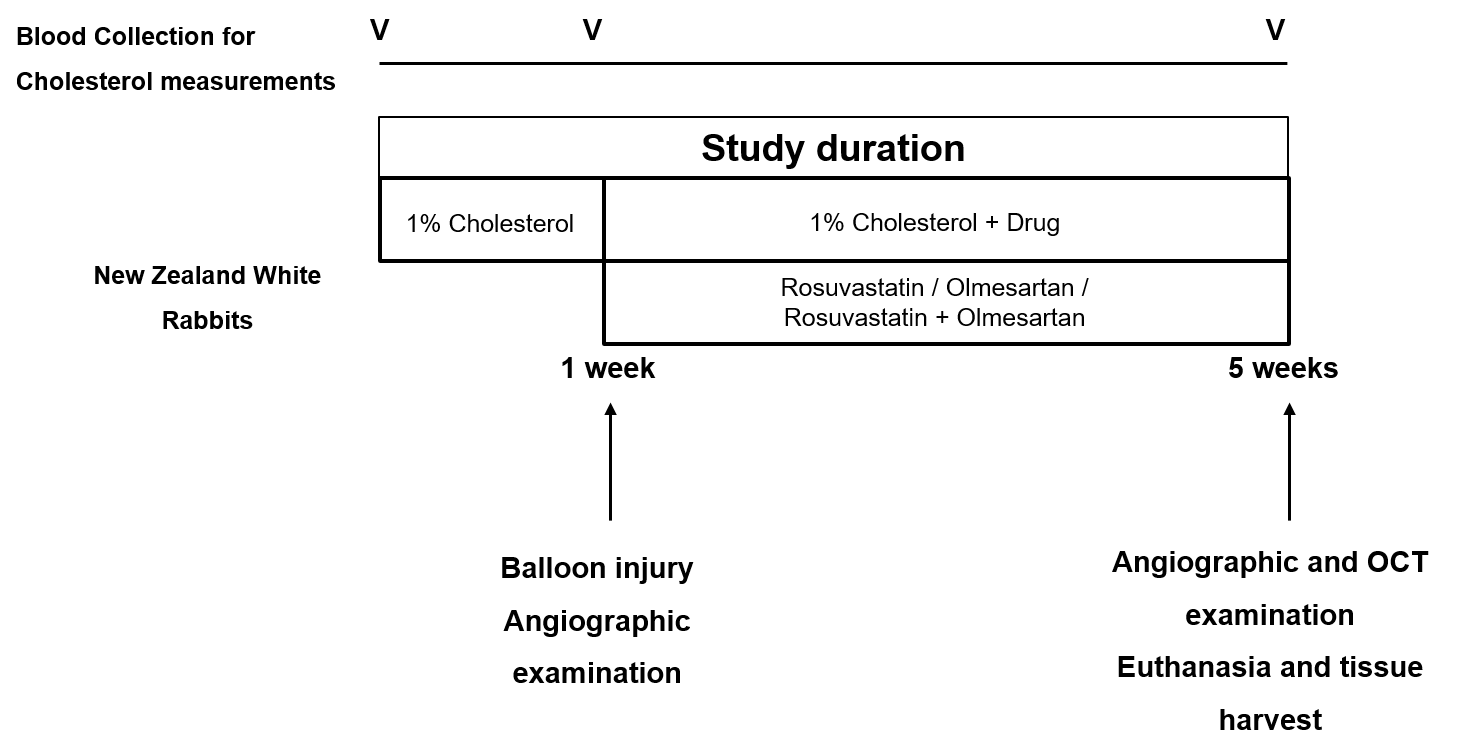

Supplement: S1 Fig — After 1 week of high-cholesterol diet, balloon injury was induced in the abdominal aortic and iliac arteries. Rabbits then received one of three drug treatments, depending on their group assignment, for 4 weeks. Blood was collected before the high-cholesterol diet and immediately before sacrifice at the end of week 5. Optical coherence tomography was assessed at the end of the study, after which the rabbits were euthanized for harvesting of their injured vessels. (TIF) [file pone.0215604.s001.tif]
