# Supplementary material for: Synergistic protective effects of a statin and an angiotensin receptor blocker for initiation and progression of atherosclerosis
Source: PLoS One. 2019 May 3;14(5):e0215604. doi: 10.1371/journal.pone.0215604 (PMC6499436; doi:10.1371/journal.pone.0215604)
Supplement: S1 Table — Values are mean±SEM. * p<0.05 vs. positive control group. ARB, angiotensin II receptor blocker; HDL, high-density lipoprotein cholesterol; LDL, low-density lipoprotein cholesterol; TC, total cholesterol; TG, triglyceride. (DOCX) [file pone.0215604.s003.docx]

|  | **Negative Control (n=3)** | **Positive Control (n=5)** | **Statin (n=5)** | **ARB (n=5)** | **Statin+ARB (n=5)** |
| --- | --- | --- | --- | --- | --- |
| **Baseline** |  |  |  |  |  |
| **TC, mg/dL** | 31.0±19.0 | 28.8±7.5 | 18.2±2.8 | 15.5±6.2 | 17.0±3.0 |
| **TG, mg/dL** | 55.7±14.3 | 40.6±8.0 | 72.4±17.4 | 77.8±11.5 | 67.8±15.1 |
| **LDL, mg/dL** | 19.7±13.4 | 4.8±1.1 | 8.1±3.5 | 9.1±2.4 | 10.4±3.2 |
| **HDL, mg/dL** | 9.0±0.0 | 20.2±7.8 | 11.8±1.1 | 9.0±3.8 | 13.8±2.8 |
| **4-weeks follow-up** | |  |  |  |  |
| **TC, mg/dL** | 20.0±3.5** | 1372.8±304.2 | 995.6±151.1 | 1053.3±214.8 | 971.8±184.5 |
| **TG, mg/dL** | 30.3±8.9 | 55.0±32.2 | 47.6±11.5 | 81.8±38.0 | 89.6±21.4 |
| **LDL, mg/dL** | 5.1±2.2** | 1459.6±245.7 | 917.9±143.5 | 999.9±312.0 | 891.7±172.1 |
| **HDL, mg/dL** | 11.0±2.5* | 80.6±17.8 | 68.2±16.6 | 49.0±10.2 | 62.2±16.5 |

**p <0.001, *p <0.01 by Dunnett post-hoc analysis
