# Supplementary material for: Synergistic protective effects of a statin and an angiotensin receptor blocker for initiation and progression of atherosclerosis
Source: PLoS One. 2019 May 3;14(5):e0215604. doi: 10.1371/journal.pone.0215604 (PMC6499436; doi:10.1371/journal.pone.0215604)
Supplement: S2 Table — Blood pressure was measured at baseline and 1-week after treatment with 20 mg/kg/day of Olmesartan in rabbits (n = 5). Values are mean±SEM. (DOCX) [file pone.0215604.s004.docx]

| **N = 5** | **Baseline** | **1 week follow-up** | **P value** |
| --- | --- | --- | --- |
| **SBP, mmHg** | 107.23±2.39 | 101.87±0.71 | 0.09 |

SBP, systolic blood pressure.
